# Perceptual Not Attitudinal Factors Predict the Accuracy of Estimating Other Women’s Bodies in Both Women With Anorexia Nervosa and Controls

**DOI:** 10.3389/fpsyg.2019.00997

**Published:** 2019-05-09

**Authors:** Lucinda J. Gledhill, Hannah R. George, Martin J. Tovée

**Affiliations:** ^1^Institute of Psychiatry, Psychology & Neuroscience, King’s College London, London, United Kingdom; ^2^Deaf Children, Young People and Family Service (National Deaf CAMHS), York, United Kingdom; ^3^School of Psychology, University of Lincoln, Lincoln, United Kingdom

**Keywords:** anorexia nervosa, body size over-estimation, body mass index, eating disorders, contraction bias

## Abstract

Disturbance in how one’s body shape and size is experienced, usually including over-estimation of one’s own body size, is a core feature of the diagnostic criteria of anorexia nervosa (AN). Is this over-estimation specific to women with AN’s judgments of their own body? Or is it just a general feature of their judgments about all bodies? If the latter, it would be consistent with a general error in the perception of body size potentially linked to the use of a different set of visual cues for judging body size. If the former, then this suggests that the over-estimation of own body size has a strong attitudinal component and may be part of the psycho-pathology of their condition. To test this hypothesis, 20 women with AN and 80 control observers estimated the body size of 46 women. The results show a strong effect of perceptual factors in estimating body size for both controls and women with AN. This result is consistent with size over-estimation of own body in AN having a strong attitudinal basis and being a core feature of the psycho-pathology of the condition.

## Introduction

A key feature of anorexia nervosa (AN) is an overestimation of body size as compared to control subjects (Collins et al., 1987; Steiger et al., 1989; Gardner and Bokenkamp, 1996; Smeets et al., 1998; Tovée et al., 2000, 2003), with women with AN consistently overestimating their own body size and having a markedly thinner ideal body size than control subjects (e.g., Williamson et al., 1993; Tovée et al., 2000). Low self-esteem, high instances of depression, a drive for “thinness,” and the media’s portrayal of a thin ideal are also suggested to contribute to body image disturbance (BID) in women (Zipfel et al., 2014; Tatangelo and Ricciardelli, 2015; Jucker et al., 2017; Moscone et al., 2017).

Body image disturbance has been shown to be one of the most persistent of all the eating disorder symptoms. Its severity seems to predict the long-term outcome of treatment (Pike, 1998; Fairburn et al., 2003). Furthermore, this persistence predicts the rate of relapse (Slade and Russell, 1973; Channon and DeSilva, 1985) which may be as high as 40% over the first 12-months post-discharge from treatment (Berkman et al., 2007; Carter et al., 2012).

Cash and Deagle (1997) suggest this disturbance in body size estimation is comprised of two components: Perceptual and Attitudinal/Cognitive. The perceptual component is described as the inability to accurately estimate body size. In contrast, the attitudinal component is described as a subject’s dissatisfaction with and negative attitudes toward their own weight and shape. Interestingly, it has been suggested that these disturbances in estimation seem specific to judgments about bodies, and do not generalize to judgments of other objects (e.g., Slade and Russell, 1973; McCabe et al., 2006). Several studies have found that this overestimation of own body size in women with AN can be seen during a variety of visual and non-visual judgments of body size (Cash and Deagle, 1997; Farrell et al., 2005; Gardner and Brown, 2014; Gaudio et al., 2014).

A possible mechanism for this pattern of response estimation is a perceptual phenomenon called contraction bias (Cornelissen et al., 2013). Contraction bias arises when one uses a standard reference or template for a class of objects (such as bodies) against which to estimate the size of other examples of that object class (Poulton, 1989). The estimate is most accurate when a given object is of a similar size to the reference but becomes increasingly inaccurate as the magnitude of the difference between the reference and the object increases. When this happens, the observer estimates that the object is closer in size to the reference than it really is. As a result, an object smaller in size than the reference will be over-estimated and an object larger will be under-estimated. Contraction bias postulates that everyone holds a mental reference for familiar stimuli, and that the effects of contraction bias are most apparent when there are no concrete units of measurement with which to judge the stimuli, such as when estimating the size of a human body.

In the case of bodies, this reference template is proposed to be based on an average of all the bodies someone has viewed over the course of their lives; with more emphasis being placed on the bodies that have been viewed most recently, i.e., the bodies of those around them and those in the media (e.g., Winkler and Rhodes, 2005; Rhodes et al., 2013). Previous studies have suggested that contraction bias predicts the accuracy of estimates of observer’s own body size (Cornelissen et al., 2015, 2016b), and is consistent with the finding that the size of obese bodies is systematically under-estimated (Kuskowska-Wolk and Rössner, 1989; Kuchler and Variyam, 2003; Maximova et al., 2008; Truesdale and Stevens, 2008: Wetmore and Mokdad, 2012; Robinson and Kirkham, 2013; Oldham and Robinson, 2015; Cornelissen et al., 2016b).

The contraction bias explanation predicts that the accuracy of body size estimation will be influenced by the BMI of the body being judged. This implies that those with AN would also overestimate the weight of other women with low BMIs, and the results of some studies are consistent with this hypothesis (Horndasch et al., 2015; Moody et al., 2017). However, when women with AN make estimates of own BMI, the pattern of body size judgments cannot simply be explained by perceptual factors. Using the same paradigm that showed a clear contraction bias effect in body size judgments by control women, when judging own body size, women with AN were accurate about making judgments of their own size when their BMI was very low but showed a very rapid increase in the magnitude of their over-estimation of their body size as BMI increased beyond the under-weight category, suggesting a significant attitudinal component specific to women with AN (Cornelissen et al., 2015, 2016b).

This rapid increase in the magnitude of body size over-estimation as their BMI moves into the normal range is potentially a contributing factor in a patient’s relapse and is consistent with the finding that the retention of body concerns is a strong predictor of relapse (Slade and Russell, 1973; Channon and DeSilva, 1985). A key additional question is whether this pattern of over-estimation of body size is specific to a woman with AN’s own body or extends to other women’s bodies as well. The accuracy of judgment of other women’s bodies plays a key role in the social comparison of the size of an observer’s body relative to their peer group, which in turn plays a role in the initiation and maintenance of eating disordered behavior (Morrison et al., 2004).

If the pattern of over-estimation when judging own BMI seen in women with AN is an attitudinal factor, we can make the prediction that judgments of other women’s bodies should not show the same pattern of over-estimation. Instead, both women with AN and controls should show the same pattern of accuracy in judgments of bodies varying in BMI. Both sets of observers should over-estimate the low BMI bodies as predicted by the perceptual phenomenon of contraction bias.

To directly answer this question, we have tested the accuracy of body size judgment of a set of 46 digital photographs of women’s bodies by controls and women with AN. This allows us to determine the accuracy with which these observers can judge a range of BMI values, and whether there are between and within group differences in the pattern of estimation.

## Materials and Methods

### Participants

The experimental procedures and methods for participant recruitment for this study were approved by the local ethics committees at Northumbria and Newcastle Universities and the Newcastle and North Tyneside Research Ethics Committee. We recruited a total of 100 women to take part in the study (see Table 1 for details). A sample of 80 controls (mean age: 26.8 years; SD: 9.4; range: 18–50 years) were recruited for this study through the undergraduate Research Participation Scheme run by the School of Psychology and the Institute of Neuroscience Volunteers scheme both at Newcastle University. Participants’ BMI ranged from 15.2 to 32.3, with a mean of 22.4 kg/m^2^. None of the control participants reported they currently had or had a history of an eating disorder. We also recruited a sample of 20 women with AN from the Richardson Eating Disorders Service at the Royal Victoria Infirmary in Newcastle upon Tyne. Inclusion criteria were only that the women had a DSM-IV diagnosis of AN, diagnosed by a senior health care professional (primarily the specialist consultant psychiatrist) and were receiving treatment at the time of the study. The participants had a mean age of 25.8 years (SD: 8.5; range: 18–46 years). And their BMIs ranged from 13.0 to 26.0 kg/m^2^, with a mean of 19.0 kg/m^2^.

**Table 1:** The means and standard deviations (*SD*) for the psychological scales for all participants.

	*N* = 100		
	Mean	*SD*	Cronbach (*α*)
Age (years)	27.24	9.06	
BMI	21.73	3.53	
Beck Depression Inventory (BDI) Max. score = 63	11.70	12.39	0.893
Rosenberg Self Esteem (RSE)	20.90	7.17	0.939
Max. score = 30			
Body Shape Questionnaire (BSQ)	46.46	18.22	0.981
Max. score = 96			
Eating Disorder Beliefs Questionnaire (EDBQ)	26.97	20.43	0.977
Max. score = 400			

### Stimuli

This study uses photographs of real women rather than the CGI body stimuli used previously (e.g., Gledhill et al., 2017; Cornelissen et al., 2018). It was thought that judgments made purely about weight would be more ecologically valid using images of real women and would avoid potential artifacts in the use of morphed simulation of adiposity in CGI bodies.

**FIGURE 1:**
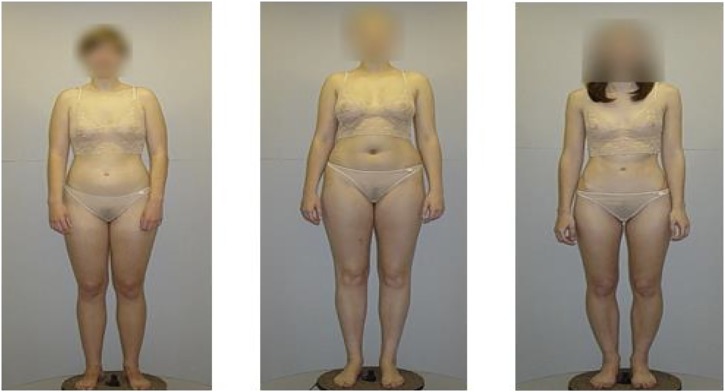
An example of the 2D forward facing images used in this study [the stimuli were collected in the Smith et al. (2007a) study].

Stimuli consisted of 46 24-bit color digital photographs of women wearing a standardized unsupportive flesh colored vest and briefs (for details of the image collection see Smith et al., 2007a). The women in the images varied in BMI from 18.3 to 26.7 kg/m^2^ (mean 22.3, SD 2.3). Faces were blurred to remove any effects of facial cues (see Figure 1).

### Materials

The Eating Disorder Beliefs Questionnaire (Cooper et al., 1997), the 16 item- Body Shape Questionnaire (Evans and Dolan, 1993), Beck’s Depression Inventory (Beck et al., 1961) and Rosenberg’s Self-Esteem Scale (Rosenberg, 1979) were used to assess attitudes toward eating and behaviors associated with Eating Disorders.

The *Eating Disorder Beliefs Questionnaire (EDBQ)* was developed as a multidimensional measure designed to assess the different types of core beliefs and assumptions held by those suffering from eating disorders. It consists of 4 subscales each designed to approach a different aspect of these assumptions: (i) negative self-belief, (ii) weight and shape as a means to acceptance by others, (iii) weight and shape as a means to Self-acceptance, and (iv) control over eating. In past studies (Cooper et al., 1997) Cronbach coefficient alphas were computed for each factor to assess their internal consistency. These values were: negative self-beliefs 0.93 (range 0.92–0.93), acceptance by others 0.94 (range 0.93–0.94), self-acceptance 0.88 (range 0.85–0.87), control over eating 0.86 (range 0.82–0.87), suggesting that these subscales all have high reliability.

The 16-item version of the *Body Shape Concern Questionnaire (BSQ)* is designed to measure concern about body shape and appearance. There are six response choices (never, rarely, sometimes, often, very often, and always) relating to how the person has been feeling over the past 4 weeks. Scores can range from 16 to 96, with high/marked concerns categorized as those with scores over 66, while those with scores less than 38 were said to demonstrate no concerns. Scores of 38–51 show mild concern while a score of 52–66 shows moderate body shape concerns (Evans and Dolan, 1993).

The *Beck Depression Inventory (BDI)* is a 21-item questionnaire designed to assess the severity of depression and was originally based on psychiatric observations of the attitudes and symptoms associated with depression. Past research found that for psychiatric populations, Cronbach’s alpha ranged from 0.72 to 0.91, with a mean of 0.86. Within non-psychiatric samples, the mean alpha was 0.81; with a range of 0.73 to 0.92, again suggesting high reliability for this questionnaire (Osman et al., 2004).

The *Rosenberg Self-Esteem Scale (RSE)* is a 10-item self-report scale where the participants are asked about general feelings about themselves and asked to tick the response closest to how the feel, with a choice of four responses (strongly agree, agree, disagree, strongly disagree). The highest total score is 30; however, Rosenberg (1979) suggests scores of 15–25 are within the normal range, whilst scores below 15 suggest low self-esteem. Past research has found Cronbach’s alpha ranging from 0.72 to 0.88 showing good reliability for this questionnaire (Gray-Little et al., 1997).

### Procedure

Volunteers were first required to read an instruction sheet and give informed consent before participating in the study. They were then given copies of the EDBQ, BSQ, BDI, and RSE to complete before the experiment began. Participants were informed that if they became uncomfortable at any stage, they could take a break or withdraw completely from the study. Participants were then asked to rate a series of 46 female bodies for body size on a Likert scale ranging from 0 to 99, with 0 representing an “emaciated” body and 99 representing an “obese” body (George et al., 2011). Participants were asked to complete body size ratings for each of the bodies. They were shown each image once. Images were shown in a randomized order which differed between participants.

E-Prime version 2.0^1^ was used to create the experiment, and each trial comprised the following sequence: A black fixation cross appeared for a period of between 1,500 and 2,500 ms. The length of this interval was randomized to prevent participants predicting when the image would appear. Next, the target image (a body) appeared for a total of 2,000 ms. Following this, the observer was reminded of the rating scale from 0 to 99 and using the keyboard (pressing keys 0–9) they made their decision. A time limit was not implemented for this rating to take place, although participants were urged to make an instinctual choice to avoid over-thinking the decision. Immediately after the rating had been made, the fixation cross appeared, and the next image was presented. This continued until all 46 images had been rated. On completion of this task, participants were given a debrief which outlined the aims and predictions of the study.

## Results

Cronbach’s α calculations were performed on the raw data for the psychometric variables revealed strong inter-rater reliability for each questionnaire, with these alpha levels consistent with the Cronbach’s results from previous studies (see “Materials and Methods” section).

A substantial and statistically significant, positive correlation was found between estimated body size and the BMI of the women in the stimulus images (*r* = 0.86, *p* < 0.0001) suggesting that participants were accurately able to estimate body size. Table 2 shows the pattern of Pearson correlations between the psychometric scores, age and BMI of the observers. Strong negative correlations were found between observer BMI and BDI score, observer BMI and EDBQ score, BSQ score and RSE, RSE score and both BDI and EDBQ score. While a strong positive correlation was found between observer BMI and observer age as well as with RSE score, observer age and RSE score, BSQ score with both BDI and EDBQ score, and BDI score with EDBQ score.

**Table 2:** The pattern of Pearson correlations between all observer variables.

	Observer BMI	Observer Age	BSQ	RSE	BDI
Observer Age	0.30^∗∗^	–	–	–	–
BSQ	−0.11	−0.02	–	–	–
RSE	0.27^∗∗^	0.20^∗^	−0.45^∗∗^	–	–
BDI	−0.41^∗∗^	−0.04	0.60^∗∗^	−0.51^∗∗^	–
EDBQ	−0.48^∗∗^	−0.08	0.75^∗∗^	−0.50^∗∗^	0.72^∗∗^

*^∗^p < 0.05; ^∗∗^p < 0.01.*

### Multivariate Statistics

We wanted to quantify the relationships between observers’ weight estimates, the BMI of the women in the stimulus images, and whether observers belonged to the Anorexic (AN) or control (CON) group of participants. Evidence consistent with contraction bias requires that the regression of weight estimates on the BMI of the women in the stimuli has a slope less than one. However, this can only be valid if both measures are reported in the same units. Therefore, to make this so, we converted both to *z*-scores. In addition, we wanted to control for any additional effects of observers’ BMI, their age and psychometric performance (i.e., BSQ, BDI, RSE, and EDBQ). To model the data, we used PROC MIXED (SAS v9.4) to build a linear mixed effect model which was optimized by ensuring that (a) any fixed effect retained in the model contributed a statistically significant reduction in –2 Log Likelihood, (b) fixed effects were retained if their Type III tests of fixed effects were significant at *p* < 0.05. The only exceptions to this were where one non-significant fixed effect comprised part of a significant two-way interaction term, in which case it was retained. In addition, we permitted individual variation at the intercept level for each observer, by including a random effect with an unstructured variance-covariance matrix. Note, we used control observers as the control when dummy coding observer groups (i.e., AN versus CON). The detailed outcome of the statistical modeling is shown in Table 3 and is illustrated in Figure 1.

**Table 3:** Linear mixed effect model parameters for predicting body size estimates.

Model Parameters	*F*-value (*df*)	*Z*-value	*p*-value	Parameter estimate	Parameter 95% CI	–2 Log likelihood
Fixed Effects						
Empty Model						13853.9
Full Model						10689.5
Image_BMI	3167.26 (1,4880)		<0.001	0.64	0.62–0.66	
Group	3.95 (1,4880)		0.047	0.15	0.0020–0.29	
Group × Image_BMI	0.78 (1,4880)		0.380	−0.020	–0.063–0.024	
EDBQ Global	20.22 (1,4880)		<0.001	0.0074	0.0041–0.011	
Random Effect						
Subject covariance		6.64	<0.001	0.093		

**FIGURE 2:**
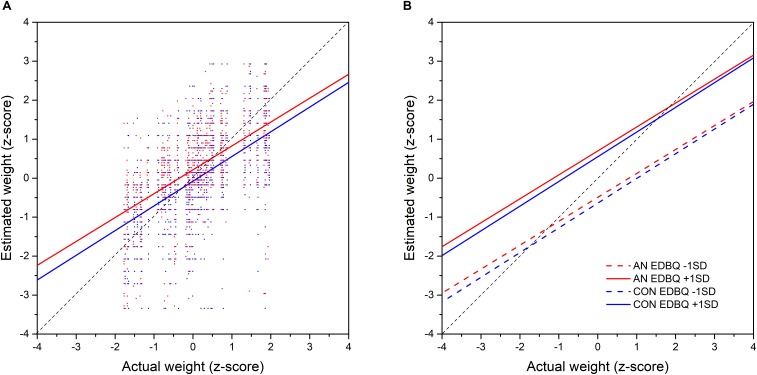
**(A)** The actual BMI of the images plotted against estimated body size of the images (*z* scored data). Red line represents the linear regression line and the black line the line equality which indicate perfect accuracy in estimation. **(B)** Fit of the body size estimations plotted against the BMI of the images. The four lines depict four groups of observers- those with low and high BMI and those with low and high psychometric scores. The black line represents the line of equality that would occur if participants were able to estimate body size perfectly.

Table 3 and Figure 2A show a statistically significant, positive relationship between estimated body weights and actual stimulus BMI. We found a marginally significant group effect: Observers with AN tended to rate stimuli as having higher body weights than did CON observers, by about ∼0.15 *z*-score units. However, there was no interaction between stimulus BMI and group. Most importantly, the slopes for the relationship between estimated weight and stimulus BMI, when expressed in *z*-scores, were significantly less than one for both groups of observers [AN: *F*(1, 44) = 44.42, *p* < 0.001; CON: *F*(1, 44) = 38.72, *p* < 0.001].

Intriguingly, we also found a statistically significant effect of EDBQ Global: higher scores on this psychometric task led to higher weight estimates in both groups of participants. This effect is illustrated in Figure 2B where the predicted values for estimated weight as a function of stimulus BMI are plotted separately for both groups at ++1 SD and –1 SD for EDBQ Global.

## Discussion

Control participants over-estimated the size of other women’s bodies at the lower end of the BMI spectrum, and under-estimated the size of the bodies at the upper end of the spectrum. This result is consistent with the perceptual phenomenon of contraction bias. Contraction bias is a feature of a particular kind of perceptual representation. The hypothesis is that complex 3D stimuli such as faces or bodies are judged in the visual system by reference to a template based on the average of all the examples of that object class that an individual has seen (Poulton, 1989). This template has multiple stimulus dimensions. For example, in the case of faces this includes nose length or the separation of the eyes and in the case of bodies it includes different aspects of body shape (Hurlbert, 2001; Winkler and Rhodes, 2005; Smith et al., 2007b; Rhodes et al., 2013). This hypothesis has been tested by selective adaptation of specific feature dimensions of this representation for both faces and bodies (e.g., Leopold et al., 2001; Rhodes et al., 2013; Sturman et al., 2017) and in the case of faces, by recording the independent modulation of neural responses along specific feature dimensions (e.g., Young and Yamane, 1992; Abbott et al., 1996; Matsumoto et al., 2005). This hypothesis suggests that the responses should show contraction bias. When making a size judgment with reference to a template a perceptual error, observers will under-estimate the size of objects which are significantly larger than the template and over-estimate the size of objects which are significantly smaller than the template (Poulton, 1989). They perceive the object as being closer in size to the reference template than it actually is. This pattern of responses has been observed in estimations of own body size by control participants in multiple studies (Cornelissen et al., 2013, 2015), and in this study is demonstrated in judgments of other women’s bodies in both control participants and in women with AN. This corpus of studies is consistent with estimation of body size by reference to a multi-dimensional template. There are of course other possible explanations for this pattern of estimation errors. One option could be serial dependence (Cicchini et al., 2017). As the name suggests, this refers to the potential impact of the previous stimulus in how the current stimulus is being rated. An image would be rated as larger if the previous image was larger and smaller if previous was smaller. Alexi et al. (2018) report the same pattern of responses in judging body size by control participants as reported here and by Cornelissen et al. (2013, 2015, 2016a,b). They interpret their results as showing serial dependency and suggest this phenomenon will help optimize the accuracy of size judgments. However, the poor quality and extreme nature of the CGI body stimuli used in this study (see Figure 4 in Alexi et al., 2018) does raise some questions as to whether the reported serial dependency effect would be observable outside the laboratory setting and the results need to be replicated using real bodies and a more realistic variation in BMI.

Some previous studies using CGI bodies have suggested that the over-estimation of body size by women with AN is primarily based on attitudinal rather than perceptual cues, particularly as the BMI of the body being judged approaches the normal range and above (Cornelissen et al., 2015, 2016b; Mölbert et al., 2017, 2018). However, this conclusion is based on each participant making an estimation of their own body size. So, each participant is only contributing only a single point to the data analysis and the estimation is based on judging bodies with BMIs similar to their own. There was no measurement of each participant’s judgments of bodies across a range of BMI values, so the *relative* accuracy of judgments across the BMI range by each participant is not known. The pattern of judgments between the BMI of the bodies and the estimation of their BMI is inferred by looking at the responses across a population of women with AN. Additionally, this judgment is of their own body size rather than making an estimation of absolute size. Furthermore, these are judgments about CGI bodies not photographs of real bodies (e.g., Cornelissen et al., 2015, 2016b; Mölbert et al., 2017, 2018; Irvine et al., 2019a,b). The studies are not using the actual variation in body size and shape that comes with changing adiposity. The adipose changes in the CGI bodies are based on the application of morphs which may be based on biometric data, but how these changes in size and shape are implemented represents a potential area of weakness which could generate experimental artifact. The current study asks participants to make a direct estimate of the body size of a set of photographs of real women varying in their BMI, to directly measure how the accuracy of estimation varies over the BMI range.

The results reported here suggest that errors in the estimations of other women’s bodies by women with AN are primarily based on perceptual factors. Lower BMI bodies are over-estimated and higher BMI are under-estimated. This is a substantial difference from the judgments of *own* body size and suggests that the psychological concerns that are proposed to determine the accuracy of own body size estimation are focused on their own body and do not produce the large-scale changes in estimation accuracy in other women’s bodies as some studies have suggested (Horndasch et al., 2015; Moody et al., 2017).

This is not to say that psychological factors play no role in the judgment of others’ body size. Although perceptual factors describe the gradient of the response between the accuracy of the estimation against the BMI of the body being judged by both the controls and the women with AN, the intercept for this relationship is also influenced by attitudinal concerns (i.e., the function moves up or down the *y*-axis depending on the magnitude of their psychological concerns as indexed by the EDBQ, see Figure 2B). This suggests that for a body of a given BMI, the magnitude of size over- or under-estimation will also be modulated by the psychological state of the observer, both in the control participants and the women with AN. So even in judgments of other women’s bodies there seems to be a significant attitudinal component to the accuracy of the size estimation.

Recent research suggests that observers are most accurate in discriminating between bodies based on size when they are presented at an angle of 45° with respect to the observer (Cornelissen et al., 2018). The data collection in the current study preceded this study and like most previous work in this area used front-view. However, as the comparison is between the size estimates made by AN and control participants and they are all judging the same sets of bodies at the same viewing angle, we believe that this comparison accurately captures any potential differences in the pattern of judgments between the two groups.

Our judgments of body size are suggested to be influenced by the sizes of the bodies we see every day both in real life and in the media (visual diet). Several studies have suggested that the exposure to larger bodies in the general population should shift our internal template toward a higher BMI, normalizing a heavier body size (e.g., Robinson and Kirkham, 2013; Oldham and Robinson, 2015). Equally, it has been suggested that the focus on thin bodies in the media and the internet (Norris et al., 2006; Ransom et al., 2010) shifts the internal template of the women with AN towards a thinner body, and so normalizes a thinner body size helping to reinforce their drive for thinness (e.g., Cornelissen et al., 2016b). Consistent with this hypothesis, cross-cultural studies have suggested a shift in ideal body size towards a preference for a lower BMI with exposure to Western media (e.g., Boothroyd et al., 2016; Thornborrow et al., 2018). Thus, any differences in accuracy of body estimation between controls and women with AN would have a perceptual basis. However, the gradient of the function between actual body size and estimated body is the same for both women with AN and controls (see Figure 2B), and the intercept difference on the *y*-axis between the two functions can be explained principally by psychological factors. This suggests that these putative differences in visual diet between controls and women with AN are not having a significant differential adaptive effect on how body size is being estimated.

The pattern of over-estimation seen in women with AN when judging their own body size seems to be incompatible with a simple perceptual explanation. This difference in between how their own body and other women’s bodies are evaluated may represent a change in how the perceptual cues to body size are appraised, or it may represent a more direct interaction between perception and cognition. For example, over-estimation of body size in women with AN and women with subclinical AN has been linked to subtle differences in how the visual information is sampled in making their judgment (George et al., 2011; Cornelissen et al., 2016a; Irvine et al., 2019a). The attitudinal concerns may create attentional biases towards specific body parts (Hewig et al., 2008; Janelle et al., 2009; von Wietersheim et al., 2012) which alters the fixation pattern used to assess body size (Cornelissen et al., 2016a; Irvine et al., 2019a). As any perceptual decision is based on the information sampled from the target, it follows logically that altering the fixation pattern will alter the perceptual judgment (Cornelissen et al., 2016a). Thus, the attentional concerns can directly alter perception. Alternatively, it may be that the errors in body size assessment are derived from the inability to assimilate perceptual information to create an accurate representation of their own body (Riva, 2018; Riva and Dakanalis, 2018). Women with AN seem to be impaired in processing global features and tend to focus on local detail (Madsen et al., 2013). This limitation in creating a holistic percept may not be limited to the visual modality, Riva has suggested impairments in women with AN may extend to integration across all sensory modalities to create an accurate personal representation and deficits in updating this representation to take into account changes in body size and shape (Riva, 2018; Riva and Dakanalis, 2018).

A key goal in treatment is to increase patient BMI into the normal range (Zipfel et al., 2015). As previously mentioned, women with AN start to increasingly over-estimate their body size as their own BMI starts to increase. If they also over-estimated the body size of other women in the same way, the apparent difference between themselves and peer-comparison of other women in the general population would be minimized. However, our results suggest that women with AN are reasonably accurate in judging the body size of other women in the normal BMI range. The result of which is to increase the size difference between their estimate of their own size and their estimate of the size of their peer-group. This peer-comparison is also likely to be a strong contributory factor, along with potential deficits in multisensory body integration, in the development and maintenance of body dissatisfaction and may play a key role in the high rate of relapse post-discharge from treatment (Berkman et al., 2007; Carter et al., 2012).

In conclusion, we note that previous studies have suggested that women with AN show an over-estimation of their own body size which cannot be explained by simple perceptual factors as seems to be the case for controls. This study found that when women with AN estimate the size of other women’s bodies, the accuracy of their estimation is primarily predicted by perceptual factors, which is consistent with the over-estimation of own body size in women with AN principally having an attitudinal rather than perceptual basis, and potentially being a psycho-pathological feature of AN.

## Ethics Statement

This study was carried out in accordance with the recommendations of the “ethics committees at Northumbria and Newcastle Universities and the Newcastle and North Tyneside Research Ethics Committee” with written informed consent from all subjects. All subjects gave written informed consent in accordance with the Declaration of Helsinki. The protocol was approved by the “ethics committees at Northumbria and Newcastle Universities and the Newcastle and North Tyneside Research Ethics Committee.

## Author Contributions

All authors planned the experiments and wrote the manuscript. LG and HG collected the data. MT analyzed the data.

## Conflict of Interest Statement

The authors declare that the research was conducted in the absence of any commercial or financial relationships that could be construed as a potential conflict of interest.

## Footnotes

1 http://www.pstnet.com
